# Effects of Vitamin D Supplementation on Cardiovascular Outcomes in Chronic Kidney Disease Patients: A Systematic Review and Meta-Analysis

**DOI:** 10.7759/cureus.87378

**Published:** 2025-07-06

**Authors:** Ayesha Saleem, Suraj S Padakanti, Mohsin Hajjaj, Muhammad Shariq Akram, Sowmya Manjari Siddenthi, Versha Kumari, Fenil Gandhi, Lakshmi Tejaswi Sakhamuri, Christopher Belletieri, Pavan K Erravelli, Ali Usama

**Affiliations:** 1 Internal Medicine, Jinnah Hospital Lahore, Lahore, PAK; 2 Internal Medicine, Ramaiah Medical College, Bangalore, IND; 3 Internal Medicine, Piedmont Macon Medical Center, Macon, USA; 4 Internal Medicine, King Edward Medical University (KEMU), Lahore, PAK; 5 Internal Medicine, Osmania Medical College, Hyderabad, IND; 6 Medicine, Abbasi Shaheed Hospital, Karachi, PAK; 7 Family Medicine, Lower Bucks Hospital, Bristol, USA; 8 Internal Medicine, Kamineni Academy of Medical Sciences and Research Centre, Hyderabad, IND; 9 Internal Medicine, Mamata Medical College, Khammam, IND; 10 Internal Medicine, Wyckoff Heights Medical Center, Brooklyn, USA

**Keywords:** ckd, function, heart, structure, vitamin d

## Abstract

Vitamin D supplementation may have beneficial effects on cardiovascular outcomes in patients with chronic kidney disease (CKD), but the underlying data are conflicting. We conducted a meta-analysis to investigate the effect of vitamin D on cardiovascular outcomes in patients with CKD.

We searched MEDLINE (via PubMed), the Cochrane Library, Scopus, and ClinicalTrials.gov from inception to 16 March 2024 for all randomized controlled trials (RCTs) assessing vitamin D supplementation in patients with CKD and reporting cardiovascular outcomes. Our primary outcomes were the incidence of adverse cardiovascular events and the change in left ventricular ejection fraction (LVEF) and left ventricular mass index (LVMI). Our secondary outcomes were the change in systolic blood pressure (SBP) and diastolic blood pressure (DBP). Data were pooled using risk ratio (RR) and mean difference as the effect measures.

A total of 11 RCTs were included in our review. Vitamin D supplementation reduced the risk of adverse cardiovascular events in patients with CKD (RR 0.39; 95% CI 0.22 to 0.69; I2=0). There was no significant difference in LVEF, LVMI, SBP, and DBP between the vitamin D and control groups.

Vitamin D supplementation does not affect adverse cardiovascular events, LVEF, LVMI, SBP, and DBP. Further large-scale RCTs and mechanistic studies are needed to understand the potential benefits of vitamin D supplementation in this patient population.

## Introduction and background

Vitamin D supplementation may have beneficial effects on cardiovascular outcomes in patients with chronic kidney disease (CKD), but the underlying data are conflicting. We conducted a meta-analysis to investigate the effect of vitamin D on cardiovascular outcomes in patients with CKD.

The global prevalence of CKD continues to rise annually, making it a significant public health concern [1]. CKD is associated with numerous complications, most notably cardiovascular disease (CVD), including heart failure and stroke. The risk of developing CVD is approximately 10 to 20 times higher in patients with CKD than in the general population [2]. Vitamin D has been proposed as a potential modulator of cardiovascular health in CKD due to its anti-inflammatory and renin-suppressive properties [2]. In patients with CKD, the production of active vitamin D (1,25(OH)₂D or calcitriol) is impaired due to a deficiency of the enzyme 1-alpha-hydroxylase, which converts 25-hydroxyvitamin D into its active form. This leads to decreased levels of vitamin D in the body, which has been linked to poor cardiovascular outcomes in patients with CKD [3]. Studies on this topic have produced conflicting results. While some investigations have suggested a favorable role of administering vitamin D in reducing cardiovascular mortality in patients with CKD [4], others have produced neutral results [5]. Certain observational studies have even indicated an increased risk of hypercalcemia and calcification of the blood vessels due to an increased amount of calcitriol in the blood. Both of these complications are associated with a high morbidity in these patients [6]. 

Given these conflicting results, we conducted a systematic review and meta-analysis to evaluate the effect of vitamin D supplementation on cardiac structure and function in patients with CKD. Specifically, we assessed changes in left ventricular mass index (LVMI) as a measure of cardiac structure and left ventricular ejection fraction (LVEF) as a marker of cardiac function.

## Review

Methods

The meta-analysis was conducted according to the Preferred Reporting Items for Systematic Reviews and Meta-Analyses (PRISMA) guidelines [7].

Search Strategy 

We used MEDLINE (via PubMed), the Cochrane Library, Scopus, and ClinicalTrials.gov from their inception to 16 September 2023 without any language limitation, using the following search string: “(Paricalcitol OR calcitriol OR Cholecalciferol OR vitamin D OR active vitamin D) AND (Left ventricular mass index OR Cardiac function OR Cardiovascular outcome OR Diastolic function OR Systolic function) AND (Chronic kidney disease OR CKD OR Nephropathy OR Kidney Failure OR Renal failure) AND (trial OR multicenter OR randomized OR placebo) NOT (review OR case report OR meta-analysis)”. We also examined the bibliographies of other systematic reviews to find further relevant articles. We used the software RevMan version 5.4.1 (RevMan International Inc., New York, USA) to make forest plots for our results.

Eligibility Criteria

**Figure 1:**
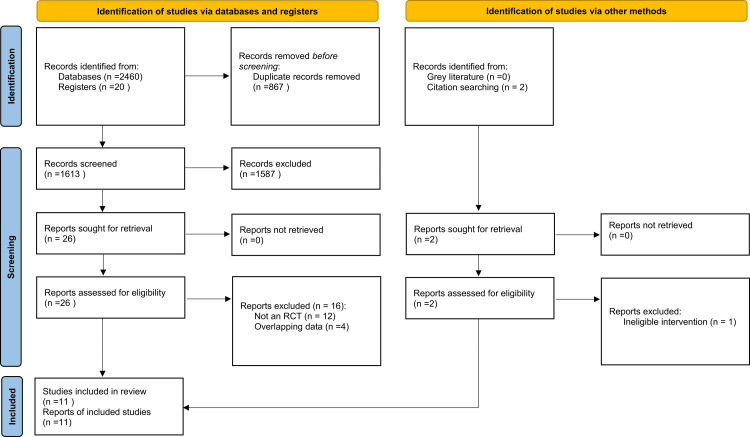
PRISMA flowchart describing systematic search and study selection process PRISMA, Preferred Reporting Items for Systematic Reviews and Meta-Analyses

Our eligibility criteria included: 1) study design: randomized controlled trials (RCTs) with a follow-up period of at least 3 months or more; 2) population: patients with established CKD, regardless of whether they were on dialysis or not; 3) Intervention: supplementation with any form of vitamin D; and 4) comparator: placebo or no treatment. We excluded observational studies, single-arm trials, and animal studies. The detailed process of inclusion and exclusion of studies is depicted in Figure 1.

Selection of Studies

After conducting the systematic search, the obtained articles were transferred to the EndNote reference library software (Clarivate, Philadelphia, USA) to identify and eliminate any duplicate sources. Our independent reviewers (AS and MH) thoroughly evaluated the remaining articles based on their titles and abstracts, followed by their full texts, and selected trials that met the eligibility criteria. A third investigator (SA) resolved any discrepancies during the review process. 

Data Extraction 

Data from the included studies were extracted into a structured Excel (Microsoft, Redmond, USA) sheet by two reviewers (AS and MH). Information pertaining to the study, population, intervention characteristics, and outcomes of interest was extracted by four additional reviewers (SP, VK, SA, and PE). Our primary outcomes were the development of adverse cardiovascular events and changes in LVMI and ejection fraction. Our secondary outcomes were the changes in systolic blood pressure (SBP) and diastolic blood pressure (DBP).

Quality Assessment 

We used the modified Cochrane risk of bias tool (RoB 2.0) to weigh the quality of the published trials. Each trial was assigned a domain-specific and overall rating of “high risk of bias,” “some concerns of bias,” and “low risk of bias.”

Statistical Analysis

For all analyses, we used the software RevMan version 5.3. We pooled the results from the trials as mean difference (MD) and risk ratio (RR), taking a CI of 95%. The Chi-square test and the Higgins I2 statistic were used to evaluate heterogeneity. To examine statistical heterogeneity, we used the Q-test for heterogeneity (Cochran 1954) and the I² statistics, with I² >50% denoting significant heterogeneity and a random-effects model otherwise. A statistically significant outcome was defined as a p-value >0.05. We applied a random-effects model for all outcomes by default, given the expected clinical and methodological diversity among studies (e.g., variations in vitamin D formulations, dosing regimens, and CKD stages). In instances of low heterogeneity (I²<25%), we cross-validated results with a fixed-effects model for sensitivity analysis; however, the random-effects model remained the primary analytic approach. A peer review by a statistician is recommended to ensure the accuracy of the model selection and the interpretation of heterogeneity metrics.

Results

After the screening process, 11 RCTs were finalized for inclusion in our study. The detailed selection process is shown in Figure 1. The trials were published between 2009 and 2023 and had follow-ups ranging between 12 and 52 weeks. Most trials used paricalcitol 1 and/or 2 μg/day, while a few administered calcitriol. The basic characteristics of the included trials are depicted in Table 1.

**Table 1:** Baseline features of the 11 RCTs compiled in this article ACEi, angiotensin-converting enzyme inhibitor; ARB, angiotensin II receptor blocker; CKD, chronic kidney disease; eGFR, estimated glomerular filtration rate; IgA, immunoglobulin A; LVEF, left ventricular ejection fraction; LVMI, left ventricular mass index; RCT, randomized controlled trial; T2D, type 2 diabetes

Lead author and year	Study population	Intervention	Primary outcome	Follow-up
Fishbane 2009 [8]	CKD, age 18–85 years, eGFR 15–90 mL/min/1.73 m2	Vitamin D1 mcg/d and placebo	Change in albuminuria: creatinine ratio	6 months
de Zeeuw 2010 [9]	Type 2 diabetes, nephropathy, age >20 years, eGFR 15–90 mL/min/1.73 m2, on ACEi or ARB	Oral vitamin D1 mcg/day, oral vitamin D2 mcg/day, and placebo	Change in albuminuria: creatinine ratio	24 weeks
Liu 2011 [10]	IgA nephropathy, age ≥18 years, eGFR >15 mL/min/1.73 m2, protein excretion >0.8 g/d, on ACEi or ARB	Oral vitamin D 0.5 mcg twice weekly and placebo	Change in 24-hour urinary protein excretion	48 weeks
Tamez 2012 [11]	CKD stages 3 and 4, eGFR 15–60 mL/min/1.73 m2	Vitamin D2 mcg/d and placebo	Left atrial volume index	48 weeks
Krairittichai 2012 [12]	Type 2 diabetes, nephropathy, age ≥18 years, eGFR >15 mL/min/1.73 m2, urinary protein creatinine ratio >1g/g	Oral vitamin D 0.5 mcg twice weekly and placebo	Change in urinary protein creatinine ratio	16 weeks
Thadhani 2012 [13]	Diverse patient population, eGFR 15–60 mL/min/1.73 m2 on ACEi or ARB, mild-to-moderate left ventricular hypertrophy	Oral vitamin D2 mcg/day and placebo	Change in LVMI	48 weeks
Joergensen 2014 [14]	Diabetic nephropathy, age 18–75 years, eGFR 15 –70 mL/min/1.73 m2	Vitamin D1 mcg/d, placebo, and then vitamin D	Urinary albumin excretion rate	12 weeks
Wang 2014 [5]	CKD stages 3 and 4, left ventricular hypertrophy	Vitamin D 1 mcg/day and placebo	Change in LVMI and LVEF	52 weeks
Lundwall 2015 [15]	CKD, age >20 years, eGFR 15–59 mL/min/1.73 m2	Vitamin D2 mcg, vitamin D1 mcg, and placebo	Blood pressure, vascular function, and glomerular filtration rate	3 months
Trillini 2015 [16]	Renal transplant	Vitamin D (1 or 2 mcg/d) and placebo	24-hour urinary protein and glomerular filtration rate	6 months
Gnudi 2023 [17]	T2D, CKD stage 3, raised left ventricular mass on ACEi or ARB	Vitamin D 0.5 mcg once daily and placebo	Change in LVMI	48 weeks

Quality Assessment 

Overall, most studies were labeled either “low risk” or “some concerns” except the Krairittichai study, which was “high risk”. The rest of the findings are summarized in Table 2.

**Table 2:** Bias risk assessment of included RCTs Coding system: (+) high risk of bias, (-) low risk of bias, (?) unclear risk of bias RCT, randomized controlled trial

Study ID	Randomization process (selection bias)	Deviations from the intended interventions (performance bias)	Missing outcome data (attrition bias)	Measurement of the outcome (detection bias)	Selection of the reported result (reporting bias)	Overall
Krairittichai 2012 [12]	_	_	+	+	?	_
Fishbane 2009 [8]	+	+	+	+	+	+
Gnudi 2023 [17]	?	+	+	+	+	?
Joergensen 2014 [14]	?	+	+	+	+	?
Liu 2011 [10]	+	?	+	+	+	+
Lundwall 2015 [15]	?	+	+	+	+	?
Tamez 2012 [11]	?	?	+	+	+	?
Thadhani 2012 [13]	+	+	+	+	+	+
Trillini 2015 [16]	?	?	+	?	+	?
Wang 2014 [5]	+	+	+	+	+	+
de Zeeuw 2010 [9]	+	+	+	+	+	+

Primary Outcomes

**Figure 2:**
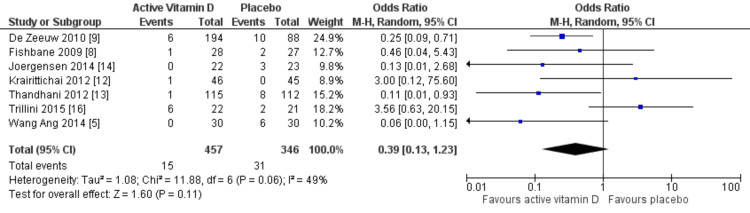
Relative risk for cardiovascular disease with vitamin D vs placebo

Effects on incident CVDs: Seven RCTs involving a total of 803 patients with CKD reported the occurrence of adverse cardiovascular events during the follow-up period. These events included hemorrhagic or ischemic stroke, acute coronary syndrome, congestive heart failure, angina, arrhythmias, and acute atrial fibrillation. Although the pooled analysis yielded an RR of 0.39 (95% CI: 0.13 to 1.23; p=0.11; I²=0), as depicted in Figure 2, suggesting a potentially favorable trend toward reduced cardiovascular risk with vitamin D supplementation, this effect did not reach statistical significance. Therefore, while the point estimate indicates a possible protective effect, it should be interpreted cautiously. No subgroup analyses were performed based on CKD stage, dialysis status, or vitamin D formulation due to limitations in the available data. Future research should investigate these subgroups to address the clinical heterogeneity among patients with CKD.

Effects on the Structure and Function of the Heart

**Figure 3:**
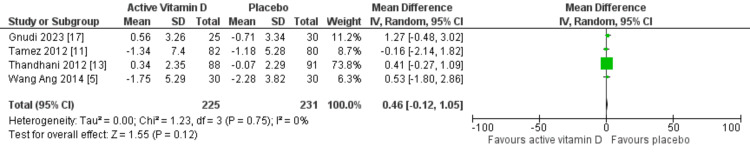
Mean difference for LVMI with vitamin D vs placebo LVMI, left ventricular mass index

**Figure 4:**
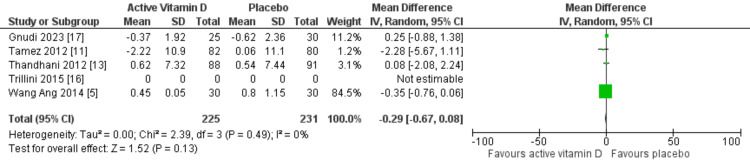
Mean difference for LVEF with vitamin D vs placebo LVEF, left ventricular ejection fraction

Cardiac structure and function were evaluated using LVMI and LVEF, respectively. Only four RCTs assessed changes in LVMI, while five RCTs reported the effects on LVEF. Our meta-analysis demonstrated that vitamin D supplementation did not significantly change LVMI (MD: 0.46 g/m²; 95% CI: -0.12 to 1.05 g/m²; p=0.12) or LVEF (MD: -0.29%; 95% CI: -0.67 to 0.08%; p=0.13), as shown in Figures 3, 4, respectively. No heterogeneity was detected in either outcome (I²=0%).

Secondary Outcome

**Figure 5:**
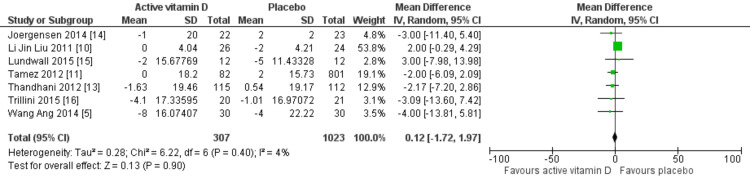
Mean difference for SBP with vitamin D vs placebo SBP, systolic blood pressure

**Figure 6:**
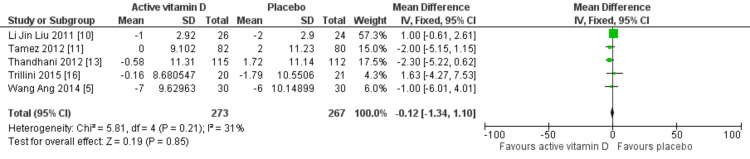
Mean difference for DBP with vitamin D vs placebo DBP, dystolic blood pressure

Effects on blood pressure: Seven RCTs evaluated the effect of vitamin D on SBP, and in all studies, patients were receiving antihypertensive therapy. The findings indicated that vitamin D treatment did not significantly reduce SBP (MD: 0.12 mmHg; 95% CI: -1.72 to 1.97 mmHg; p=0.90) or DBP (MD: -0.12 mmHg; 95% CI: -1.34 to 1.10 mmHg; p=0.85). Heterogeneity was 4% for SBP and 31% for DSP. These results are illustrated in Figures 5, 6.

Discussion

CKD, a serious medical condition, is characterized by the progressive loss of renal function. CVD is a major and closely related complication that frequently occurs in individuals with CKD. These patients are more likely to develop acute coronary syndrome, heart failure, arrhythmia, stroke, and venous thromboembolism [18]. Our meta-analysis of 11 RCTs suggests that vitamin D administration neither decreases the risk of adverse cardiovascular outcomes nor improves LVEF, LVMI, SBP, or DBP.

The findings of our study are consistent with previous research on this topic. For example, a study by Bhan et al. [19] showed that vitamin D supplementation does not produce any statistically significant reduction in cardiovascular events in patients with CKD. Similarly, Mehrotra et al. [20], in their analysis of patients with CKD from the Third National Health and Nutrition Examination Survey (NHANES III), a nationwide probability sample of 39,695 individuals conducted from 1988 to 1994, found that while low serum vitamin D was associated with higher mortality, it had no significant association with cardiovascular events in patients with CKD undergoing dialysis.

The primary endpoint outcomes included the incidence of cardiovascular events and measures of cardiac structure and function. Our meta-analysis showed no significant role of vitamin D administration in altering cardiovascular event incidence, LVMI, or LVEF. Similarly, a study by Sonkar et al. [21] found no association between vitamin D levels and cardiac structure or function in non-diabetic individuals with CKD. That study identified serum phosphate and intact PTH levels as more significant prognostic indicators for increased left ventricular mass and early diastolic dysfunction. In another study, Banerjee et al. [22] reported no beneficial effects of vitamin D on left ventricular mass or LVEF. Other studies have also demonstrated no effect of vitamin D supplementation on left ventricular mass [23,24].

Secondary endpoint outcomes included SBP and DBP. Our analysis showed that vitamin D administration does not significantly lower either measure in patients with CKD. These findings align with a study by McMullan et al. [25], which reported no blood pressure benefit from correcting vitamin D deficiency. However, a recent study by Martin-Romero et al. [26] observed reductions in aortic and brachial pulse pressures following vitamin D administration in stage 3 and 4 patients with CKD. Similarly, Cheung et al. [27] found reduced SBP in patients with CKD treated with a combination of magnesium and vitamin D supplements. These conflicting findings highlight the need for additional RCTs to conclusively determine the impact of vitamin D on blood pressure.

There are several limitations to our study that should be acknowledged. Although statistical heterogeneity was low, variations in the enrolled populations and disease severity across the included trials may have introduced undetected clinical heterogeneity. Additionally, the duration of follow-up varied between studies, which could contribute to methodological heterogeneity. Long-term outcomes could not be assessed, as no RCTs provided data beyond 52 weeks. Furthermore, the current evidence regarding the association between vitamin D and adverse cardiovascular outcomes in CKD and end-stage kidney disease (ESKD), also known as end-stage renal disease (ESRD), remains limited and inconclusive [28]. ESKD represents the most advanced stage of CKD, where the kidneys can no longer adequately filter waste and excess fluids, leading to toxin accumulation in the body.

Therefore, more well-designed clinical trials with extended follow-up periods are needed to provide conclusive evidence on the potential cardiovascular benefits of correcting vitamin D deficiency in this population. Further research is also warranted to investigate the underlying mechanisms, as the adverse cardiovascular events observed in our meta-analysis were not mediated through changes in LVEF, LVMI, or blood pressure, suggesting that other pathways may be involved.

## Conclusions

Our meta-analysis demonstrated that vitamin D supplementation did not show a statistically significant reduction in the incidence of adverse cardiovascular events in patients with CKD. Similarly, no significant effects were observed on LVMI, LVEF, SBP, or DBP. These findings suggest that current evidence is insufficient to support the routine use of vitamin D for cardiovascular protection in patients with CKD. We acknowledge several limitations of this analysis. The included RCTs varied in follow-up duration, vitamin D formulations, dosages, and patient characteristics, introducing methodological heterogeneity that may affect the generalizability of results. Most trials enrolled pre-dialysis patients with CKD, and very few included those with ESKD, also known as ESRD--the most advanced stage of CKD in which kidneys can no longer maintain homeostasis. As a result, the relevance of our findings to the ESKD population remains uncertain.

In addition, no RCT reported outcomes beyond 52 weeks, limiting the ability to assess the long-term cardiovascular effects of vitamin D supplementation. Therefore, future research should include large-scale, well-powered RCTs with extended follow-up, stratified by CKD stage (including ESKD), baseline vitamin D status, and cardiovascular risk. Studies should also explore specific formulations and dosages of vitamin D and aim to identify underlying mechanistic pathways linking vitamin D metabolism to cardiovascular health in CKD. Addressing these gaps is essential to provide clearer clinical guidance on vitamin D supplementation in this population.
